# Feasibility of Integrating Residential Care Pharmacists into Aged Care Homes to Improve Quality Use of Medicines: Study Protocol for a Non-Randomised Controlled Pilot Trial

**DOI:** 10.3390/ijerph15030499

**Published:** 2018-03-12

**Authors:** Nicole McDerby, Mark Naunton, Alison Shield, Kasia Bail, Sam Kosari

**Affiliations:** 1 Faculty of Health, Discipline of Pharmacy, University of Canberra, Bruce CBR 2617, Australia; mark.naunton@canberra.edu.au (M.N.); alison.shield@canberra.edu.au (A.S.); sam.kosari@canberra.edu.au (S.K.); 2 Faculty of Health, Discipline of Nursing, University of Canberra, Bruce CBR 2617, Australia; kasia.bail@canberra.edu.au

**Keywords:** pharmacist, residential care, medication review, dosage form modification, influenza vaccination

## Abstract

Older adults are particularly susceptible to iatrogenic disease and communicable diseases, such as influenza. Prescribing in the residential aged care population is complex, and requires ongoing review to prevent medication misadventure. Pharmacist-led medication review is effective in reducing medication-related problems; however, current funding arrangements specifically exclude pharmacists from routinely participating in resident care. Integrating an on-site clinical pharmacist into residential care teams is an unexplored opportunity to improve quality use of medicines in this setting. The primary objective of this pilot study is to investigate the feasibility of integrating a residential care pharmacist into the existing care team. Secondary outcomes include incidence of pharmacist-led medication review, and incidence of potential medication problems based on validated prescribing measures. This is a cross-sectional, non-randomised controlled trial with a residential care pharmacist trialled at a single facility, and a parallel control site receiving usual care and services only. The results of this hypothesis-generating pilot study will be used to identify clinical outcomes and direct future larger scale investigations into the implementation of the novel residential care pharmacist model to optimise quality use of medicines in a population at high risk of medication misadventure.

## 1. Introduction

### 1.1. Medication Use in Older Adults

Prescribing in the older population is highly complex. Age-related pharmacokinetic and pharmacodynamic changes lead to variations in drug bioavailability, increased drug sensitivity, and decreased regulatory mechanisms, altering the effects of drug usage from those observed in younger populations [1]. In addition, the presence of multiple co-morbidities necessitating multiple medication usage equates to an increased risk of medication misadventure in older adults [1,2]. Advancing age is positively correlated with increased prevalence of chronic disease, and increased number of co-morbidities correlates with increased medication use [3]. 

Older people residing in residential aged care homes (RACHs) have additional social and organisational factors which further complicate medicines use. These factors can include complex social and care responsibility arrangements, a high dependency on staff with minimal and variable formal medication administration training, in an environment associated with high turnover and casualised workforces, and highly variable levels of family participation and resident autonomy in treatment decision-making [4,5]. RACH residents are also the highest users of medications, with an estimated Australian average of seven medications per resident [6]. Polypharmacy is the term used to describe multiple medicines use, and a broadly accepted definition is the concurrent use of five or more medicines [3]. Polypharmacy is the most positive predictor for adverse drug events, and is associated with poor health outcomes in older adults [3,6,7]. 

### 1.2. Impact of Inappropriate Polypharmacy

Although polypharmacy may be clinically appropriate in multi-morbid patients [8], the association between inappropriate polypharmacy and iatrogenic disease is well documented in the literature [7,9]. Adverse drug events (ADEs) are any undesirable event resulting from a medication that interferes with the desired outcome, and include adverse reactions, interactions, inappropriate medications, inappropriate dosing, inappropriate administration, over or under prescribing [1,3,10]. ADEs can significantly impair occupational and cognitive functioning, and quality of life [9]. 

All medications have the potential to cause an ADE, particularly in older adults, as a result of pathophysiological decline, inappropriate polypharmacy, and involvement of multiple health providers. This can worsen cognitive impairment, frailty, disability, frequency of falls, and mortality [3,6,9]. A recent systematic review highlights a number of factors which both increase resident risk of exposure to a medication error, and increase the risk of harm resulting from medication error, including inadequate communication, inappropriate polypharmacy and lack of onsite pharmacy services [4]. Pharmacists are medicine experts, and therefore should be strategically placed to provide ongoing review to aid in the optimisation of pharmacotherapy. 

### 1.3. Current Clinical Pharmacist Roles in Residential Aged Care

The Australian residential medication management review (RMMR) program is in place for accredited pharmacists to review RACH residents at risk of medication misadventure [3,11]. Accredited pharmacists have achieved a postgraduate accreditation in medication review, and their referral-based role is shifted from that of the pharmacists’ traditional dispensing role to a collaborative and clinical service [3]. The RMMR service has been funded by the Commonwealth Government through the Department of Ageing and Aged Care since 1997, and was implemented to enable general practitioner (GP) and accredited pharmacist collaboration to optimise quality use of medicines within the aged care setting [3,12,13]. 

The efficacy of pharmacist medication review in identifying and reducing potential medication related problems is well documented in the literature, both in Australia and internationally, with various studies and reviews indicating that pharmacist medication review improves the quality of medicine use [9,14,15,16,17,18,19,20,21]. Although medication reviews are an effective intervention for identifying and resolving potential medication problems, the RMMR service, which is the primary funded service for these reviews under the National Medicines Policy in Australia, is associated with logistical difficulties and access restrictions in place, which highlight the scope for improvement. 

Face-to-face discussion of RMMR findings between pharmacists, GPs, and RACH nursing representatives via case conferencing is recommended; however, this is funded only for the referring GP and not the other stakeholders, and is not mandated under the current service model [10,22]. When they do occur, case conferencing RMMR findings are associated with higher rates of uptake of recommendations made by pharmacists during medication reviews compared with written communication of findings [10,23]. Despite this, case conferencing rates for RMMRs are reportedly less than 50% of RMMRs conducted [12]. The logistical difficulties of arranging a time for all stakeholders, including residents or their enduring power of attorney (EPOA), to be available to case conference RMMR findings, as well as the additional unpaid time for the pharmacist and RACH representative under the current funding arrangement for the service, may contribute to poor uptake of case conferencing. Further, due to the consultancy based nature of RMMRs, pharmacists lack a thorough understanding of organisational regimes and in-depth knowledge of the resident, which limits the RMMR pharmacists’ ability to contextualise recommendations to align with each specific resident and each specific organisation. 

In terms of access restrictions, RMMRs are only remunerated for residents who have resided at a RACH for a minimum of 14 days, and RACH residents are only entitled to one RMMR every 24 months, unless specific clinical criteria are present, which may qualify the resident for an earlier review [22]. These clinical criteria include discharge from hospital within previous four weeks, change in medical condition, or significant change in medication regimen within the previous three months [3]. These access restrictions may result in delays or missed opportunities for pharmacists to review residents at risk of medication misadventure.

### 1.4. Transitions of Care and Adverse Drug Events

Transitioning into aged care has been identified as a particularly high-risk point where residents are vulnerable to medication errors and ADEs [24,25,26,27]. Transitions of care for residents include new admission from the community or hospital to a RACH, or returning to the RACH post-discharge from hospital. 

Poorly executed care transitions and miscommunication can result in interrupted continuity of care and adverse events, which may lead to inappropriate re-admission to hospital or presentation to emergency departments [24,26,27]. Approximately 20% of residents experience a significant delay in medication administration and missed doses following admission or re-admission to a RACH [27]. Transition-related medication errors are observed in 13–31% of RACH residents, often involve high risk medications, such as warfarin, insulin, psychoactive agents, and opioids, and have greater risk of causing serious harm to the resident [4]. 

The importance of medication reconciliation by a pharmacist has been highlighted in the literature in reducing medication errors during transitions of care. A systematic review published in 2012 reported that pharmacist-led medication reconciliation at the point of transfer to and from residential care likely improves outcomes for residents [28]. 

In addition to hospital transition challenges for RMMR, there is also a significant problem for new admissions to RACHs, as residents must reside at a RACH for 14 days before they are eligible for a RMMR under the current access restrictions described under Section 1.3. Therefore, this high-risk period on admission to a RACH is currently excluded from the RMMR service, yet has been demonstrated to be a key time point for which medication reviews would be beneficial to minimise the risk of medication errors and to support communication, education, appropriate administration practices, and adjustments to a new medication regime post-discharge.

### 1.5. Communicable Disease Prevention

Another key role of pharmacists in RACHs is in relation to vaccination. Residents of RACHs are particularly vulnerable to morbidity and mortality associated with vaccine preventable communicable diseases, such as influenza [29]. Influenza is largely preventable through annual vaccination, and there is sufficient evidence to support RACH staff vaccination to protect residents from influenza [29,30,31]. Despite being recommended by public health organisations as an important source of prevention, RACH staff vaccination rates against influenza remain suboptimal, with as few as 28% of RACH staff being vaccinated [32]. Reasons reported by RACH staff for not receiving annual influenza vaccination include not recommended by the employer/organisation, did not know it was needed, affordability, inconvenient, or missed receiving it [32]. 

Pharmacists have been able to vaccinate people against influenza in Australia since 2015 [33]. The rational for this is to improve accessibility to the vaccination for members of the community who have difficulty accessing the vaccine through their GP or employer, as pharmacies are often open later and on weekends [33]. Investigating whether including a pharmacist in RACH staffing arrangements increases staff influenza vaccination rates is of interest to public health. 

### 1.6. Residential Care Pharmacists as an Alternative Model of Pharmacy Practice

Having an on-site residential care pharmacist (RCP) model may address some of the areas for improving care in RACHs highlighted above. The integrated RCP role differs from that of the RMMR pharmacist by facilitating more frequent face-to-face collaboration between the pharmacist and existing care team, greater understanding of resident-specific medication management decisions, and greater understanding of site-specific operational policies and procedures. Offering an on-site pharmacy service, as opposed to a visitational role currently provided by RMMR pharmacists, will enable the RCP to more efficiently follow up with residents, GPs, nursing, and care staff involved in resident care, as required. On-site integration can also aid in the development of the rapport necessary to establish the assessment of complex illnesses for complex residents, including behavioural and psychological symptoms of dementia, and to develop the trust and communication, necessary in peer relationships for carers and nurses, to share the implementation of the medication plan. This may be particularly the case for complex and titrated medication regimes, such as pain management and palliative care. This kind of communication support, and the kind of “as-required” follow up and reinforcement or adjustment approach, is not currently funded under the current RMMR service model. 

A RCP model that is integrated into the RACH allied health team may improve collaboration and case conferencing opportunities for GPs, nursing staff, and pharmacists. Additionally, a RCP model may enable more opportunities for including the resident or their EPOA as part of a patient-centred medication review model, by circumventing the logistical issues associated with arranging case conferencing between multiple visiting clinicians. To date, the impact of a RCP model on case conferencing opportunities and resident/carer inclusion in the RMMR process has not been reported in the current literature. 

Currently, there is no available data investigating the impact of an on-site RCP model on resident outcomes during transitions of care. This highlights an important area for potential intervention to reduce medication errors for new admissions to RACHs and for returning residents post-discharge from hospital by expediting access to medication review. 

Given that pharmacist-led influenza vaccination is a relatively recent addition to pharmacy practice, there are no publications reporting the impact of a pharmacist-led influenza vaccination program within RACHs to improve staff vaccination rates. 

It is important that this novel role is explored, to challenge the current landscape of medicines use in RACHs considering the aging population, increasing co-morbidity rates, and intensifying medical management of disease.

### 1.7. Aim

The aim of this pilot study is to investigate the feasibility of integrating an on-site RCP model into an established RACH. This project is designed as hypothesis-generating research, rather than hypothesis-testing, and thus will be used to identify potential clinical and operational outcomes for further investigation. 

## 2. Materials and Methods 

### 2.1. Objectives

The primary objective of this pilot study is to investigate whether it is feasible to integrate an on-site RCP into residential aged care. This will be examined through evaluation of RACH staff and resident/EPOA perceptions, and resident quality use of medicines. 

The secondary objectives of this pilot project will be to describe the activities performed by the RCP, and identify various clinical and operational opportunities that may fall under the scope of this new pharmacist role, warranting further investigation. The secondary objectives for this pilot study are listed under Table 1.

### 2.2. Design

This is a cross-sectional, non-randomised controlled trial that will include an intervention period of six months, with a three-month pre-intervention and three-month post-intervention observation period. Baseline data from the six-month period preceding the intervention period will be collected during the pre-intervention phase, and data from the six months during the intervention will be collected during the post-intervention phase for comparison. Table A1 provides the Standard Protocol Items: Recommendations for Interventional Trials (SPIRIT) Figure utilised in the design of the schedule of enrolment, intervention, and assessments [34]. This hypothesis-generating pilot study utilises an ethnographic approach to explore the feasibility of integrating a clinical pharmacist into RACHs. 

### 2.3. Setting

This pilot controlled study will be conducted in two RACHs located in the Canberra region of Australia. Both sites belong to the same organisation, operating under similar procedural guidelines, staffing ratios, and organisational culture, with equitable access to external health professionals, including GPs, nurse practitioners and allied health services. Staffing and management ratios are similar. The first site has 104 residential beds, is staffed by a total of 70 nursing and care staff, and will serve as the intervention site where the RCP will pilot the new pharmacist role. The second RACH has 100 resident capacity, and is staffed by 80 nursing and care staff, and will serve as the parallel control site receiving usual care and services only. Nursing and care staff are site-specific and not routinely shared between the two RACHs. The site where the RCP role is to be trialled will be nominated by the organisation.

### 2.4. Participants

All residents, GPs, nursing and care staff at the intervention and control RACHs will be invited to participate in this pilot study. Participation in the study will be open to all residents and care staff, regardless of duration of residence/employment at the RACHs or cognitive status. The only criterion for exclusion from the study is declining to provide written consent for participation. Consent for participation will be provided by the EPOA for residents with a dementia diagnosis documented in their medical history at the RACH. Carers and people with EPOA for the resident with dementia may have a complementary or alternate perspective to the person with dementia, hence both are included for broad and representative information collection about the service, and to facilitate shared decision-making [35]. 

Both residents and RACH staff are free to withdraw their consent to participate at any stage during the pilot study. Resident baseline data collected during the pre-intervention and post-intervention phases will have a re-identifiable code, so that data can be removed upon request. Staff survey responses and interviews will be non-identifiable, so cannot be removed from the data set once submitted. These terms are described in the participant information form. 

All participants are required to give their informed consent for inclusion before they participate in the study. The study was designed in accordance with the Declaration of Helsinki, and the protocol was approved by the University of Canberra Human Research Ethics Committee (HREC 16–244) on the 22nd of December 2016.

### 2.5. Sample Size 

The intervention site has 104 resident capacity, and the control site has 100 resident capacity. The intervention site has 78 staff and the control site has 104 staff, including nursing, administration, cleaning, catering, and care staff. This study is a hypothesis-generating pilot project; thus power calculations are inappropriate for the primary outcome and majority of secondary outcomes. However, a sample size power calculation looking at the secondary outcome of improving staff vaccination rates was performed to establish whether RACH staff numbers are sufficient to indicate a potentially significant increase in staff influenza vaccinations. Using staff influenza vaccination data from the intervention site in 2016 and a 95% confidence interval, there are sufficient staff (*n* = 78) to power statistical significance for an increase in staff influenza vaccination rates from 46% in 2016 to 69%, at both RACHs, in 2017.

### 2.6. Recruitment Strategies

Residents at the intervention and control RACHs will be recruited over a period of nine months. Residents and their EPOAs will be invited to participate in the study by the RACH care manager for the duration of the pre-intervention phase (−*T1*), three months prior to the commencement of the intervention, and continue with new RACH admissions, invited to participate until the conclusion of the intervention. This study aims to recruit above 50% of residents at each site to the study. 

RACH staff, including nurses, carers, allied health, and GPs, will be recruited over a period of twelve months, commencing from the pre-intervention phase (−*T1*), and concluding at the end of the post-intervention phase (*T1*), three months after the intervention phase has concluded. Staff at both sites will be invited to participate in the study, and provide qualitative data to support findings in the pre-intervention, intervention, and post-intervention phases. 

The pharmacist recruited for the RCP role during this project will be the accredited pharmacist who holds the RMMR contract for the facilities. The RCP will be expected to continue their usual role and RMMR practice models outside the trial hours at both the control and intervention sites, as the RCP is being tested as an additional service, rather than replacement service, as the role is in the early stages of development. The pharmacist will be registered with the Australian Health Practitioner Regulation Agency (AHPRA) and the Australian Association of Consultant Pharmacy (AACP), and have substantial experience in conducting RMMRs. The pharmacist will have professional indemnity insurance as required by the Pharmacy Board of Australia. 

### 2.7. Intervention

The intervention is to integrate a clinical pharmacist into the existing care team at an established RACH. An accredited pharmacist experienced in RMMRs will be piloting the role of RCP at the intervention site for a period of six months. The RCP will work at the intervention site for two consecutive days, totalling 15 h per week (0.4 full time equivalent) for six months. The RCP’s role will require formal and informal integration into the existing care team at the facility, working closely with the nursing and management teams to improve quality use of medicines for residents and medication-related operational efficiencies for staff. 

The RCP will introduce the role to the GPs with patients at the intervention RACH, and be responsible for developing the role and professional relationships with RACH care staff, nursing staff, and other allied health professionals, including physiotherapy and occupational therapy. The RCP will also be responsible for managing the workflow associated with the role, and prioritise tasks as appropriate to the needs of the residents and organisation. 

The intervention is within a registered and accredited pharmacist’s scope of practice, providing medication reviews, staff influenza vaccinations, and quality improvement activities. The intervention role is an evolution of the current RMMR pharmacist’s role, further integrated into the RACH clinical team. A medication management consultation service will still be provided to both sites. 

### 2.8. Outcomes

The primary and secondary outcomes for this study have been identified using the Grading of Recommendations, Assessment, Development, and Evaluations (GRADE) methodology [36]. The primary outcome of this pilot study will be to determine the feasibility of the RCP role in the RACH setting by exploring the views and experiences of stakeholders, including care staff, GPs, residents, community pharmacists, and RCP involved in the project. 

Seventeen secondary outcomes have been identified for the proposed intervention role compared with usual standards of care, and are described in Table 2.

### 2.9. Data Collection

Data addressing the primary and secondary outcomes will be collected and collated by the principle investigator from iCareHealth software, operational audit records, survey and interview responses, and the RCP activity records, to address the primary and secondary outcomes. Data will be collected at baseline (*T0*) and following conclusion of the intervention (*T1*), and entered into a database and stored on a password protected device. Staff at both sites will be invited to participate in surveys which will investigate usual interactions with pharmacy services. Medication rounds and electronic administration records will be audited to obtain baseline data to inform the secondary project outcomes. Post-intervention data (*T1*) will be used for comparison to baseline data (*T0*) within each facility, as well as comparison between the intervention and control site, to determine whether there are any outcomes which may indicate benefit from having a RCP integrated into RACHs.

The RCP will record all activities undertaken at the intervention RACH on a purposefully designed electronic recording form. The activity record forms will be submitted to the principle investigator weekly for collation into a database, and analysis. Discussion regarding data collection and observations regarding the RCP experience will contribute to the ethnographic capture of components and outcomes of the pilot. The activity records will describe

1. The activity that was performed, the time taken to perform the activity, and whether the activity was completed on the same day.
2. The outcomes or benefits to the resident involved and/or staff as perceived by the RCP.
3. Any potential adverse outcome avoided by completing the activity as perceived by the RCP.
4. The person who requested the activity, if any, and who else would complete the activity if the RCP had not.
5. The unique re-identifying code for the resident(s) involved in the activity if applicable.

RCP activities will be classified into six major categories and twenty subcategories, described under Table 3. The categorisation coding value is indicated for each subcategory. The frequency of activity categories documented by the RCP will indicate the types of pharmacist interventions that are frequently utilised in the RACH setting, and the time spent providing these services.

A communication diary for staff to leave requests and enquiries for the RCP to follow up will be provided to the facility and collected at the end of the intervention phase. The RCP will document any interventions in the resident notes, as deemed clinically appropriate, to inform care staff and physicians. 

The research team will collect feedback on the acceptability of the RCP role, as well as the benefits and barriers of the RCP role as perceived by participating residents, EPOAs, nursing and care staff, allied health, GPs, and the pharmacist piloting the role. This feedback will be collected during the post-intervention phase (*T1*) through paper-based surveys and short semi-structured interviews. Participation in either feedback method will be optional for all stakeholders.

### 2.10. Statistical Analysis

Quantitative and qualitative data will be obtained to explore the RCP role in terms of the primary and secondary outcomes. Descriptive statistics will be used to summarise the RCP activity data, resident demographic data, and secondary outcome data at the conclusion of the post-intervention period (*T1*). Parametric tests, such as ANOVA, chi-square, and *t*-tests will be used for the results, with normal distribution, non-parametric testing to be used for any data with skewed distributions. All statistical tests will be two-tailed with a *p*-value set at 0.05. The intervention arm will be compared with the control arm at baseline (*T0*) and post-intervention (*T1*) for changes relating to the secondary outcomes. SPSS Statistics 22.0 software (IBM, Armonk, NY, USA) will be used for all quantitative statistical analysis. Depending on the survey response rate, responses may be analysed using regression test. Qualitative data derived from transcribed interviews and open-ended survey questions will undergo thematic analysis, with NVIVO 11.0 software (QSR International, Melbourne, Australia) used to supplement organisation and filing. 

### 2.11. Data Monitoring

Residents at both the control and intervention RACHs will remain under the care of their usual GP at all times during the pilot study. The GPs associated with the facilities are not included in the research team, and will therefore serve as independent reviewers to assess and report any adverse events during the project that may be related to the intervention. Any suspected adverse event associated with the RCP intervention will be managed with the GP and care facility to support the resident health in the first instance, and reported to the research team and Human Research Ethics Committee for review. 

## 3. Discussion

There are anticipated practical limitations for this pilot study associated with the part-time nature of the role and presence of the RCP on site. These include delayed follow up of interventions where required, and difficulty timing presence for face-to-face meetings and case conferencing with visiting GPs and family members. This may impact the time taken to discuss and implement changes recommended by the RCP as a result of medication review. Depending on the timing of administrative policy meetings, such as the Medicines Advisory Committee meeting at the facility, the RCP may not be able to attend on the designated schedule, and thus miss opportunities to implement positive changes at the facility-wide policy level. Conversely, attending these meetings and implementing policy changes adopted by the organisation may lead to medication management improvements at the control facility. A controlled study design enables testing of RCP feasibility between the comparable sites, however, the risk of contamination is present, due to the intervention and control sites being run by the same organisation. 

The patient population recruited for this study are frail older adults with multiple co-morbidities, including dementia, who may have limited life expectancy, terminal illness, or require acute hospital treatment for non-medicine related causes. In conjunction with the small population size anticipated for this pilot study, demonstrating a statistically significant decrease in secondary clinical objectives, such as reducing hospital admissions, length of hospital stay, reduced emergency department presentations, or mortality, will be unlikely. Further, this study is measuring the incidence of staff training and education sessions, not to evaluate whether such sessions translate to a successful impact on behaviour. This would be a useful addition to future studies, but is outside the scope of this trial. 

## 4. Conclusions

The results of this study will be useful for stakeholders in the health and aged care sectors to inform further investigation and possible decision-making about service values. The implementation of a new model for the delivery of clinical pharmacist services, in a setting where patients with complex health needs remain at high risk from medication misadventure, will also be useful to refine future empirical evidence in this burgeoning gerontological area. The clinical and operational insights gained from this study will inform as to whether the integration of a RCP into RACHs is feasible, and may inform potential government or private industry funding models to support the role of the RCP as part of the clinical team within RACHs. 

## Figures and Tables

**Table 1:** Secondary clinical and operational objectives.

Clinical Objectives	Operational Objectives
Optimise rational use of medications	Optimise staff influenza vaccination rates
Improve rate of pharmacist-led medication reviews	Quality improvement in medicines management
Reduce frequency of hospital admissions	Policy development
Reduce length of stay in hospital	Optimise collaboration between clinicians & carers
Reduce frequency of emergency department presentations	Optimise time taken to correct medication errors on new/readmission to residential aged care home (RACH)
Support optimisation of pharmacotherapy in collaboration with prescribers, residents and carers	Undertake point of care testing for residents requiring ongoing monitoring
Improve implementation of pharmacist recommendations made during medication reviews	Provide opportunities for on-site medicine education
Reduce falls	Source of drug information
Reduce medication refusals	

**Table 2:** Secondary clinical and operational outcomes.

Outcome	Measure
1. Clinical	
1.1. Potential medication related problems	The Drug Burden Index is a validated measure of exposure to sedative and anti-cholinergic medicines [37,38]. The Medication Appropriateness Index is a validated standardised instrument for assessing drug therapy [39]. Both tools will be used to determine the incidence of potential medication related problems in resident medication regimes at baseline in both control and intervention residents. These tools will be re-applied following the intervention, to determine whether there is a reduction in the incidence of potential medication related problems at the intervention site, and use the control site to determine whether any reduction in the incidence of potential medication related problems at the intervention site is attributable to the residential care pharmacist (RCP).
1.2. Pharmacist-led medication reviews	Baseline incidence and reason for residential medication management reviews (RMMRs) will be collected from both sites and compared with rates and reason of pharmacist-led medication reviews (both RMMRs and RCP-led reviews) during the intervention period, to determine whether the frequency of review rates increased for residents, and whether the reason for medication review was routine or based on clinical need.
1.3. Implementation of pharmacist recommendations	The rate of implementation of recommendations made by the RCP following medication reviews conducted during the intervention period will be compared with the rate of implementation of recommendations made by pharmacists following RMMRs at both sites before and during the intervention. This will be used to identify whether there is a difference in the rate of acceptance and implementation of recommendations made by pharmacists in these two review models.
1.4. Hospital admission	The incidence of resident hospital admissions at both sites will be recorded pre- and post-intervention, to determine whether the RCP has an impact in reducing hospital admissions by reducing medication-related problems.
1.5. Length of hospital stay	The incidence of average length of hospital stay for residents at both sites will be recorded pre- and post-intervention, to determine whether the RCP has an impact in reducing length of hospital stay by preparing and improving medication profiles and information transferred with the resident to hospital.
1.6. Emergency department presentations	The incidence of resident emergency department presentations at both sites will be recorded pre- and post-intervention, to determine whether the RCP has an impact in reducing presentations to emergency departments by reducing medication related problems.
1.7. Falls	The incidence of resident falls at both sites will be recorded pre- and post-intervention, to determine whether the RCP has an impact in reducing falls by reducing medications taken which increase residents risk of falls.
1.8. Medication refusals	The incidence of resident medication refusals at both sites will be recorded pre- and post-intervention, to determine whether the RCP can reduce the incidence of resident medication refusals by rationalising medication regimes. The medications that are refused will also be documented at both sites to potentially identify classes of medications that are refused by residents.
1.9. Correction of medication errors on new admissions to RACH	Time spent correcting medication errors for residents who are new admissions to the intervention RACH will be recorded in the RCP activity data. This will determine the frequency that this service is utilised, and the types of errors identified. Quantitative and qualitative data derived from surveys and interviews will be used to support whether this service is valued and utilised by RACH staff.
1.10. Correction of medication errors following discharge from hospital	Time spent correcting medication errors for residents who are transferring back to the intervention RACH, following discharge from hospital, will be recorded in the RCP activity data. This will determine the frequency that this service is utilised, and the types of errors identified. Quantitative and qualitative data derived from surveys and interviews will be used to support whether this service is valued and utilised by RACH staff.
2. Operational	
2.1. Staff influenza vaccination	Staff influenza vaccination rates from 2016 to 2017 will be provided by the RACHs, and will be compared within each site, as well as compared between intervention and control sites to determine whether having an on-site pharmacist vaccination service improves rates of staff influenza vaccination.
2.2. Provision of medicine-related training and education for staff	Incidence of staff training and education sessions in medicine management by a pharmacist during the intervention will be recorded in the RCP activity data. Training will be available to all levels of staff involved in medication administration, including all levels of nursing staff and care staff. This will determine the frequency that this service is utilised, and quantitative and qualitative data derived from surveys and interviews will be used to support whether this service is valued and utilised by RACH staff.
2.3. Collaboration between clinicians and carers	Incidence of collaborative interactions with prescribers, RACH staff, residents, and carers will be recorded in the RCP activity data and qualitative data. These interactions will include case-conferencing between stakeholders, interactions supporting appropriate deprescribing of medicines, and providing medicines’ information to residents. This will determine the frequency that this service is utilised, and quantitative and qualitative data derived from surveys and interviews will be used to support whether this service is valued and utilised by all stakeholders. Collaborative practice will be considered in relation to the Pharmacist Code of Conduct [35].
2.4. Pharmacist-led point of care testing	Incidence of resident point of care testing performed by the RCP including: blood pressure, blood glucose, and international normalised ratio will be documented through the RCP activity data to determine the frequency that this service is utilised within the intervention RACH.
2.5. Provision of drug information and pharmaceutical opinion	Time spent providing drug information and pharmaceutical opinion for residents and care staff within the RACH will be recorded using the RCP activity data, including who else would provide this information in the absence of the RCP, to determine the frequency that this service is utilised within the intervention RACH. Pharmaceutical opinion is the term used to classify activities where the RCP supplied advice on therapeutic management of a resident, but was not a comprehensive review of the resident’s medication management, and may or may not involve direct resident contact. Drug information is the term used to classify activities where the RCP supplied information on a medication without any resident-specific context. Quantitative and qualitative data derived from surveys and interviews will be used to support whether this service is valued and utilised by RACH staff.
2.6. Inappropriate dosage form modification	Pre-intervention observational audits of medication rounds will obtain baseline rates of inappropriate solid dosage form modification (tablet crushing) at both facilities. A second round of observational audits of medication rounds will be conducted post-intervention, to identify any decrease in rates of inappropriate solid dosage form modification.
2.7. Quality improvement in medicines handling	Pre- and post-intervention comparison of audits on medication storage, medication administration, schedule 8 medication handling, and medication ordering processes will be used to evaluate areas of impact by the RCP.

**Table 3:** Categorisation scheme for RCP activities.

Major Activity Category	Activity Subcategories
1. Medication review	1.1. Comprehensive medication review [Time; minutes]
	1.2. Pharmacotherapy optimised [Yes/No]
	1.3. New admission [Yes/No]
	1.4. Post-hospital discharge [Yes/No]
	1.5. Risk focussed assessment [Yes/No]
	1.6. Health assessment [Yes/No]
2. Communication	2.1. Resident interaction [Yes/No]
	2.2. GP interaction [Yes/No]
	2.3. Community pharmacy interaction [Yes/No]
	2.4. Nurse/other RACH staff interaction [Yes/No]
3. Education	3.1. Staff training [Time; minutes]
	3.2. Resident education [Time; minutes]
	3.3. Drug information [Yes/No]
	3.4. Pharmaceutical opinion [Yes/No]
4. Quality Improvement	4.1. Audit [Time; minutes]
	4.2. Quality improvement activity [Time; minutes]
5. Vaccination	5.1. Staff vaccination [Yes/No]
	5.2. Resident vaccination [Yes/No]
6. Administration	6.1. Project meeting [Time; minutes]
	6.2. RACH policy meeting [Time; minutes]

## Appendix A

**Table A1:** The Standard Protocol Items: Recommendations for Interventional Trials (SPIRIT) Figure for the schedule of enrolment, intervention, and assessments. *T0* baseline, *T1* for 3 months following completion of intervention.

Study Procedures	Study Period			
	**Enrolment**	**Post-Allocation**		**Post-Intervention**
**TIMEPOINT**	***−T1***	***T0***	***Intervention***	***T1***
**ENROLMENT:**				
Eligibility screen	X			
Written informed consent	X			
Site Allocation	X			
**INTERVENTIONS:**				
Residential Care Pharmacist			X	
Usual care			X	
**ASSESSMENTS:**				
Baseline data for study outcomes		X		
Baseline RACH staff surveys		X		
RCP activity data			X	
Follow up data for study outcomes				X
Follow-up RACH staff surveys				X
Resident/EPOA surveys				X
Interviews with RACH staff				X

*T1* 3 months prior to intervention; *T0* baseline; *T1* 3 months following completion of intervention.
